# Potent In Vitro and In Vivo Effects of *Stachys lavandulifolia* Methanolic Extract against *Toxoplasma gondii* Infection

**DOI:** 10.3390/tropicalmed8070355

**Published:** 2023-07-05

**Authors:** Abdullah D. Alanazi, Qais A. H. Majeed, Sultan F. Alnomasy, Hamdan I. Almohammed

**Affiliations:** 1 Departmentof Biological Sciences, Faculty of Science and Humanities, Shaqra University, P.O. Box 1040, Ad-Dawadimi 11911, Saudi Arabia; 2 Department of Science, College of Basic Education, PAAET, Aridiya, Kuwait City 23167, Kuwait; qa.majeed@paaet.edu.kw; 3 Department of Medical Laboratories Sciences, College of Applied Medical Sciences, Shaqra University, Al-Quwayiyah 19257, Saudi Arabia; s.alnomasy@su.edu.sa; 4 Department of Basic Science, Faculty of Medicine, Almaarfa University, Riyadh 11597, Saudi Arabia; hamohammed@mcst.edu.sa

**Keywords:** *Stachys lavandulifolia*, toxoplasmosis, RH strain, in vivo, in vitro, herbal medicines

## Abstract

The present study aimed to evaluate the in vitro, in vivo, and safety of *Stachys lavandulifolia* Vahl. methanolic extract (SLME) against acute toxoplasmosis caused by *Toxoplasma gondii* RH strain in mice. Methods: MTT (3-(4,5-Dimethylthiazol-2-yl)-2,5-diphenyltetrazolium bromide) assay was used to evaluate the in vitro effect of the SLME on *T. gondii* tachyzoites. Totally, 72 male BALB/c mice (40 mice for in vivo evaluation of SLME and 32 mice for its toxicity effects on liver and kidney serum enzymes) were used for the present investigation. At first, 40 mice were orally pre-treated with the SLME at doses of 25, 50, and 75 mg/kg/day for two weeks. Mice were checked daily, and the rate of survival and the mean number of tachyzoites were recorded. Liver lipid peroxidation (LPO) and nitric oxide (NO) levels, the effects on kidney and liver function, as well as the expression level of the proinflammatory cytokines such as interleukin-1β (IL-1β) and interferon-γ (IFN-γ), were studied by the quantitative real-time PCR. Flow cytometry analysis was performed on the effects of SLME on the detection of apoptotic and necrotic cells in *T. gondii* tachyzoites. Results: The SLME at the concentrations 75 and 150 µg/mL completely killed the tachyzoites after 2 hr of incubation. SLME at 25, 50, and 75 mg/kg/day increased the survival rate of infected mice by the sixth, seventh, and eighth days, respectively. SLME also significantly (*p* < 0.05) decreased the LPO and NO levels and upregulated the IL-1β and IFN-γ mRNA gene expression levels, whereas no considerable change was observed in the serum level of kidney and liver enzymes. Flow cytometry analysis revealed the prompted early and late apoptosis after exposure to *T. gondii* tachyzoites with various concentrations of SLME. Conclusion: We found the relevant in vitro anti-*Toxoplasma* effects of SLME against *T. gondii*. Moreover, the results confirmed the promising in vivo prophylactic effects of SLME. SLME provokes the innate immune system, induces apoptosis, modulates the proinflammatory cytokines, and inhibits hepatic injury in infected mice. With all these descriptions, further surveys are required to support these findings and elucidate this plant’s possible mechanisms of action.

## 1. Introduction

Toxoplasmosis caused by *Toxoplasma gondii* is a prevalent parasitic disease between humans and animals [1]. According to seroepidemiological studies, up to 30% of the world’s population is infected with this parasite and has antibodies against this parasite in their blood circulation [2]. Toxoplasmosis is usually transmitted by eating food and water contaminated with oocysts and eating tissue cysts in undercooked meat [3]. On the other hand, if placental transmission of parasites (tachyzoite forms) occurs during pregnancy, it can cause congenital toxoplasmosis in the fetus [4]. The common form of toxoplasmosis in immunocompetent healthy individuals is usually asymptomatic and resolves spontaneously, whereas in some people, the infection occurs as spontaneous lymphadenopathy, and in a few cases, as cerebral and ocular symptoms [5]. Among the host cell responses, cell-mediated immune responses through the production of the proinflammatory cytokine, e.g., interleukin 12 (IL-12), IL-1β, and IFN-γ, by macrophages and dendritic cells (DCs) are considered as the main modulating inflammatory pathways for controlling of parasite burden and disease in toxoplasmosis [6].

Today, the main strategy for treating the acquired and congenital forms of toxoplasmosis and even toxoplasmosis in people with weakened immune systems is chemotherapy with a combination of pyrimethamine and sulfadiazine along with several synthetic agents such as atovaquone and spiramycin, etc. [7]. There are reports that these drugs have serious side effects, such as increasing the risk of bone marrow suppression, hematological toxicity, teratogenic effects, and renal complications [7]. Therefore, finding effective drugs with minimal side effects and optimal effectiveness in the treatment of toxoplasmosis is necessary.

Currently, a number of studies reported the prophylactic ability of various medicinal herbs, e.g., *Zataria multiflora*, *Allium sativum*, *Cinnamomum zeylanicum*, *Curcuma longa*, *and Berberis vulgaris* [8], but the clinical use of these medicinal herbs to prevent toxoplasmosis has not been supported because of different conclusions and their safety.

Among the medicinal herbs, the genus *Stachys* is considered one of the main and most broadly recognized genera of the Lamiaceae family and comprises 258 genera and around 7000 species worldwide [9]. *Stachys lavandulifolia* Vahl. is a perennial herbaceous plant that is grown mostly in Saudi Arabia, Syria, Iraq, and Central Asia [9]. In traditional medicine, *S. lavandulifolia* is a famous herbal tea used to relieve pain and is a sedative for treating gastrointestinal disorders and infections, etc. [10]. Moreover, this plant, due to having a high amount of phenolic and terpenoid compounds, has various pharmacological properties such as wound healing, antibacterial, antiviral, and antiparasitic effects [11].

Although several studies have been carried out on the anti-*Toxoplasma* effects of medicinal herbs, nevertheless, their findings have been diverse and every so often inconsistent, which could affect the efficacy of the medicinal herbs because of the type of extraction, part used, parasite strain, and the method of assay [12,13]. 

Since the beneficial effects of the *Stachys* spp., such as the neuroprotective, hepatoprotective effect, anti-inflammatory, reducing oxidative stress (antioxidant), anti-nociceptive, etc., have been proven, the present study aimed to evaluate in vitro, in vivo, and safety of the *S. lavandulifolia* methanolic extract (SLME) against acute toxoplasmosis caused by *T. gondii* RH strain in mice.

## 2. Materials and Methods

### 2.1. Plant Collection

*S. lavandulifolia* leaves were procured from a market in Riyadh City, Saudi Arabia, and the materials were identified at the Department of Biological Science, Shaqra University. The herb herbarium samples (no. 50-2021) are preserved in the Department of Biological Science, Shaqra University, Saudi Arabia.

### 2.2. Ethics

The Ethical Committee for Animal Experiments of Almaarefa University, Saudi Arabia (no. IRB07-18052022-46) approved the current work.

### 2.3. Preparing of Extract

Two hundred-fifty grams of the dried whole leaves were extracted through a percolation procedure with methanol (70%) consecutively for 3 days at 21 °C. In the next step, it was passing the extracts through filter paper (Sigma, Roedermark, Germany), and lastly, was evaporated in a vacuum at 50 °C utilizing a rotary evaporator (Heidolph, Germany) and kept at −20 °C until testing [14,15].

#### 2.3.1. Phytochemical Analysis 

The primary phytochemical screening of the SLME was carried out to assess the presence of tannins, saponins, alkaloids, flavonoids, and terpenoids based on previous investigations [16,17]. 

#### 2.3.2. Quantity Analysis of the Secondary Metabolites

Folin–Ciocalteau’s assay was utilized to determine the total content of phenolic (TCP) compounds [18]. In brief, SLME (20 μL) was added to the distilled water Folin–Ciocalteau (100 μL) solution. Then, 0.3 mL sodium carbonate liquid (20%) was added to the mixture, and its absorbance was measured at 760 nm. TCP was reported in terms of milligrams of gallic acid per gram of extract. For determining the total flavonoid content (TFC) of SLME, aluminum chloride solution (0.1%) was added to potassium acetate (0.1%), ethanol (95%), and distilled water and incubated at 21 °C for 30 min. Then, the absorbance of the combination was read at 415 nm. TFC was reported in terms of milligrams of quercetin per gram of extract [19].

### 2.4. Parasites

In this study, virulent *T. gondii* RH strain tachyzoites were kindly provided from the King Saud University of Medical Science, Riyadh, Saudi Arabia. Parasites were kept through serial intraperitoneal passages in BALB/c mice. After three days, tachyzoites were obtained and centrifuged at 21 °C for discarding artifacts and peritoneal cells. Tachyzoites were filtered via 3.0 μm pore-size filters (Nuclepore) and washed in phosphate-buffered saline (PBS) and then were adjusted into 1 × 10^6^ and 1 × 10^4^ parasite per each mL by a hemocytometer slide for in vitro and in vivo tests, respectively [20].

### 2.5. Animals

Totally, 72 male BALB/c mice (40 mice for in vivo evaluation of SLME and 32 mice (aged 6 to 8 weeks) for its toxicity effects on liver and kidney serum enzymes) with weights between 20 and 25 g were designated for the present investigation. 

### 2.6. In Vitro Anti-Toxoplasma Effects

The MTT (3-(4,5-Dimethylthiazol-2-yl)-2,5-diphenyltetrazolium bromide) assay was used to evaluate the in vitro effect of the SLME on *T. gondii* tachyzoites [20]. In summary, 200 µL of tachyzoites (1 × 10^6^ cells/mL) were incubated with 200 µL of SLME at the concentrations of 32.5, 75, and 150 µg/mL (the selection of these concentrations was based on the primary experiments) at 37 °C for 0.5, 1, 2, and 3 h. Normal saline + Tween 20 (as solvent) and atovaquone 30 µg/mL [20,21] were considered as the negative and positive control. Followed by the incubation time, and after adding 50 µL of the MTT solution (5 mg/mL), the tested tubes were again kept warm at 37 °C for 240 min with 5% CO_2_. Lastly, the dimethyl sulfoxide solution (DMSO) as a stopping solution was added to the test tubes to dissolve the crystals of formazan. The absorbance of the tested microplate was measured at 630 nm by an ELISA reader LX800 (Bio-Rad, Hercules, CA, USA). The experiments were performed in triplicate. The half maximal inhibitory concentration (IC50) was also calculated for SLME and atovaquone.

#### 2.6.1. Flow Cytometry Analysis 

Here, to study the effects of SLME on the detection of apoptotic and necrotic cells in *T. gondii* tachyzoites, we used the Annexin-V Kit (BioVision, Waltham, MA, USA) based on the producer’s instructions. In summary, tachyzoites (1 × 10^6^ cells/mL) were incubated with SLME at 32.5, 75, and 150 µg/mL in 24-well plates for 48 h at 24 °C. Followed by centrifuging at 2500 rpm and discarding the upper phase, we added the kit materials, including binding buffer, Annexin-V58T, and propidium iodide (PI) to the wells, and were incubated in dark condition for 5 min at 21 °C. Finally, absorbance in cells was measured at the excitation wavelength of 570 nm and the emission wavelength of 630 nm by flow cytometry (Sysmex Partec GmbH, Munster, Germany), and the obtained findings were examined by FlowJo software.

#### 2.6.2. Effect of Intracellular Parasites and Infectivity Rate

To evaluate the effects of SLME on intracellular parasites, at first, Vero cells (10^5^ cells/mL) were exposed to tachyzoites (1 × 10^6^ cells/mL) at the ratio of 1:10 at 37 °C for 24 h. The infected cells were exposed to SLME at 32.5, 75, and 150 µg/mL for 180 min. To determine the effects of SLME on infectivity rate, tachyzoites (1 × 10^6^ cells/mL) were initially exposed to SLME (32.5, 75, and 150 µg/mL) for 4 h at 24 °C, then, Vero cells (10^5^ cells/mL) was exposed to treated tachyzoites at 37 °C for 24 h. Following the preparation of the slides, they were stained by Giemsa and examined by a light microscope to determine the infectivity rate and the number of intracellular parasites by testing 100 infected cells [20].

#### 2.6.3. Cytotoxicity Effects of SLME on Normal Cells 

Briefly, SLME at 50, 100, 200, 400, and 800 µg/mL were separated into the 96-well microplate containing Vero cells (1 × 10^6^ cells/mL) for 48 h at 37 °C with 5% CO_2_. Like assessing of in vitro anti-*Toxoplasma* effects of SLME, we used an MTT assay to study the cytotoxicity effects of SLME on normal cells. The 50% cytotoxic concentration (CC50) was also calculated for Vero cells exposed to SLME [22,23].

### 2.7. In Vivo Anti-Toxoplasma Effects

Forty male BALB/c mice were separated into five groups (GP) (8 mice per each), including GPi: vehicle control group, orally administrated with the normal saline; GPii: positive control group, orally received atovaquone 100 mg/kg/day [24]; GPiii: orally received SLME at 25 mg/kg/day for 14 days; GPiv: orally received SLME at 50 mg/kg/day for 14 days; and GPv: orally received SLME at 75 mg/kg/day for 14 days [21,24].

Then, 24 hrs after the end of the treatment (on the 15th day), 1 × 10^4^ tachyzoites were inoculated intraperitoneally to each mouse. The survival rate of tested mice was recorded by daily checking of all 40 mice tested. Furthermore, the parasite load was determined on the 3rd day post-infection by calculating the mean number of tachyzoites in the peritoneal fluids of all 40 mice tested using a light microscope [20]. After aspiration of peritoneal fluids (1 mL), it was centrifuged for 5 min at 200 *g* at 21 °C to discard artifacts and peritoneal cells. In the next step, the supernatant was discarded, and the remaining tachyzoites were recovered with PBS and were calculated by a hemocytometer slide. In addition, three mice from each group were euthanized (by intraperitoneal injection of the sodium pentobarbital at 150 mg/kg [24]) to study the LPO, NO, and proinflammatory cytokines.

#### 2.7.1. Evaluation of Liver Lipid Peroxidation (LPO) and Nitric Oxide (NO)

Malondialdehyde (MDA) in liver homogenates was measured as an indicator of lipid peroxidation based on its reaction with thiobarbituric acid (TBA) according to the protocol of the commercial kit (Abcam, ab118970). In addition, the effect on the production of NO in liver homogenates was studied based on the protocol of the commercial Nitric Oxide Assay Kit (abcam, ab65328) (ab118970). 

#### 2.7.2. Proinflammatory Cytokines mRNA Expression

The mRNA expression level of the proinflammatory cytokines of interleukin-1β (IL-1β) and interferon-γ (IFN-γ) in liver homogenates was determined by the quantitative real-time PCR. To do this, by using a RNeasy tissue kit (Qiagen, Hilden, Germany), total liver RNA was extracted. Next, we used random primers to synthesize the complementary DNA (cDNA) for real-time PCR via the SYBR green. The oligonucleotide primers used in real-time PCR were F: 5′AACCTGCTGGTGTGTGACGTTC3′, and R: 5′CAGCACGAGGCTTTTTTGTTGT3′ for IL-β, F: 5′ATGAACGCTACACACTGCATC3′ and R: 5′CCATCCTTTTGCCAGTTCCTC3′ for IFN-γ, and F: 5′GTGACGTTGACATCCGTAAAGA3′ and R: 5′GCCGGACTCATCGTACTCC3′ for β-actin (housekeeping gene). The thermal condition of the reaction was 96 °C for 5 min, 40 cycles of 96 °C for 10 s, and 57 °C for 30 s, respectively. Lastly, the ΔCt was determined using the iQTM5 optical system software (Bio-Rad, Hercules, CA, USA) [25].

#### 2.7.3. Toxicity Effects on Liver and Kidney Functions 

For evaluating the toxicity profile of the SLME on liver and kidney function, 32 remaining healthy mice were separated into four groups (HGP) (8 mice each), including HGPi: vehicle control group, orally administrated with the normal saline; HGPii: orally received SLME at 25 mg/kg/day for 14 days; HGPiii: orally received SLME at 50 mg/kg/day for 14 days; and HGPiv: orally received SLME at 75 mg/kg/day for 14 days [21,24]. The day after two weeks of treatment, through the cardiac puncture of mice, the blood specimens were collected via sterile syringe with and lacking the anticoagulant (EDTA). After centrifuging the blood samples at 5000 rpm for 10 min, the obtained serums were stored at −20 °C. Finally, kidney and liver biochemical factors such as creatinine (cr), blood urea nitrogen (BUN), alanine aminotransferase (ALT), aspartate aminotransferase (AST), alkaline phosphatase (ALP), and bilirubin (direct and total) were assessed utilizing the commercial diagnostic kits [26,27]. 

### 2.8. Statistical Analysis

All the in vitro examinations were carried out in triplicate. The obtained data were displayed as the means ± standard deviation. SPSS statistical software version 22.0 (SPSS Inc., Chicago, IL, USA) was applied for data analysis. The difference among experimental groups was studied by using one-way ANOVA with Tukey’s post hoc. *p* < 0.05 was statistically significant.

## 3. Results

### 3.1. Phytochemical Analysis

The phytochemical analysis of the *S. lavandulifolia* methanolic extract exhibited the attendance of a high level of flavonoids, tannins, alkaloids, and terpenoids and a lack of saponins in this plant (Table 1). The analysis and measurement of the contents of secondary metabolites displayed that the total flavonoid and phenolic content was 19.58 mg quercetin equivalents per gram of dry weight (mg QE/g DW) and 29.53 mg of gallic acid equivalents per gram of dry weight (mg GEA/g DW).

### 3.2. In Vitro Anti-Toxoplasma Effects

**Figure 1:**
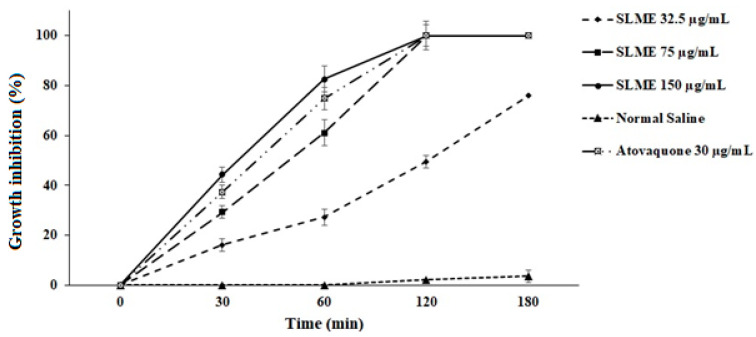
In vitro activity of different concentrations of the SLME on the growth inhibition of *T. gondii* tachyzoites after 0.5, 1, 2, and 3 h treatment. Results are displayed as the mean ± SD. The experiments were performed in triplicate.

Based on the obtained results of the MTT assay, different concentrations of SLME exhibited significant (*p* < 0.001) antiparasitic effects against *T. gondii* tachyzoites after 0.5, 1, 2, and 3 h exposure in comparison with the control group. As shown in Figure 1, SLME at the concentrations of 75 and 150 µg/mL completely killed the tachyzoites after 2 hr incubation. In addition, 100% mortality was observed after 3 hr incubation of tachyzoites with SLME at the concentration of 75 µg/mL. The IC_50_ value for SLME after 30, 60, 120, and 180 min was >150, 61.3, 32.7, and <32.5 µg/mL, respectively, whereas these values for atovaquone were 133, 66.7, <32.5, and <32.5 µg/mL, respectively.

### 3.3. Flow Cytometry Analysis

**Figure 2:**
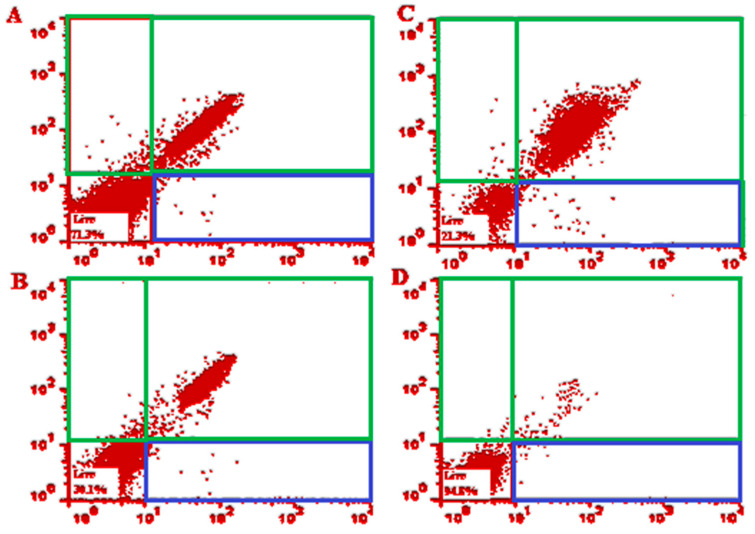
The effects of *Stachys lavandulifolia* methanolic extract at 32.5 μg/mL (**A**), 75 μg/mL (**B**), 150 μg/mL (**C**), and normal saline + Tween 20 as solvent (**D**) on the level of apoptotic and necrotic cells in *Toxoplasma gondii* tachyzoites, by flow cytometry analysis. (i) Early apoptosis; (ii) late apoptosis; (iii) necrotic cells.

The obtained findings of flow cytometry revealed the prompted early and late apoptosis after exposure to *T. gondii* tachyzoites with various concentrations of SLME. As shown in Figure 2, the early apoptosis after exposure to SLME at 32.5, 75, and 150 μg/mL was 0.640%, 1.23%, and 1.72%, respectively, whereas the late apoptosis after exposure to SLME at 32.5, 75, and 150 μg/mL was 27.5, 67.8%, and 76.7%, respectively.

### 3.4. Effect of SLME on Intracellular Parasites, Infectivity Rate, and Cytotoxicity

**Figure 3:**
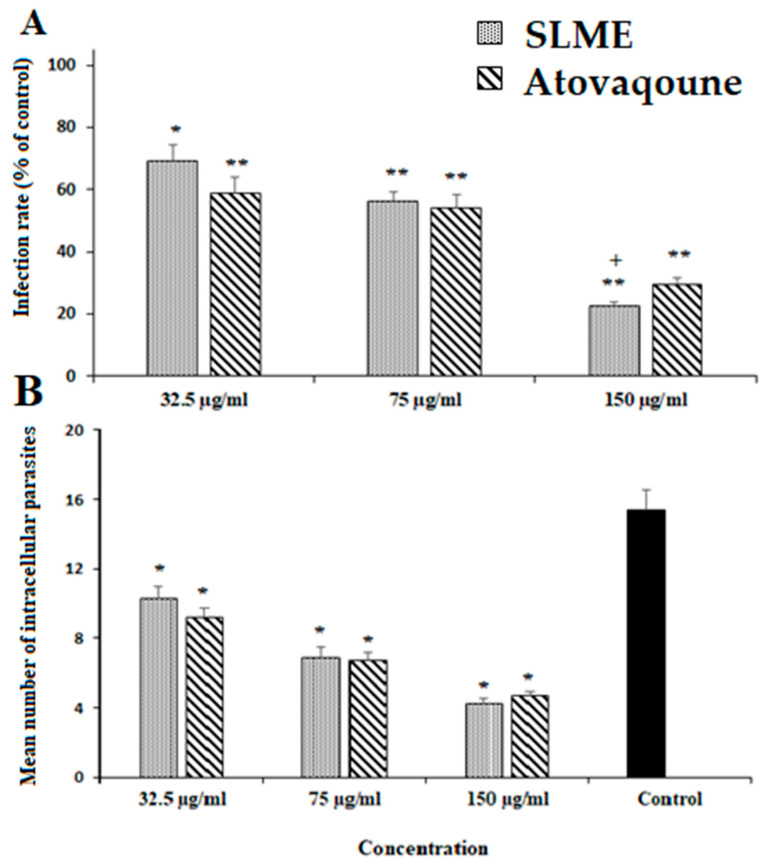
Effects of *Stachys lavandulifolia* methanolic extract (SLME) and atovaquone (30 μg/mL) on infectivity rate (**A**) and intracellular parasites (**B**). (n = 3). * *p* < 0.05; ** *p* < 0.01 compared with the control; + *p* < 0.05 compared with atovaqoune.

As depicted in Figure 3, SLME declined the infectivity rate of the infected cells in a dose-dependent manner, whereas at the concentration of 150 µg/mL markedly decreased (*p* < 0.001) by 22.6%. The intracellular multiplying of parasites in infected cells was also markedly inhibited (*p* < 0.001) after exposure to SLME (Figure 3). Based on the results of the MTT assay, the CC_50_ value for ECEO and atovaquone was 489.4 μg/mL and 435.2 μg/mL, respectively.

### 3.5. In Vivo Anti-Toxoplasma Effects

**Figure 4:**
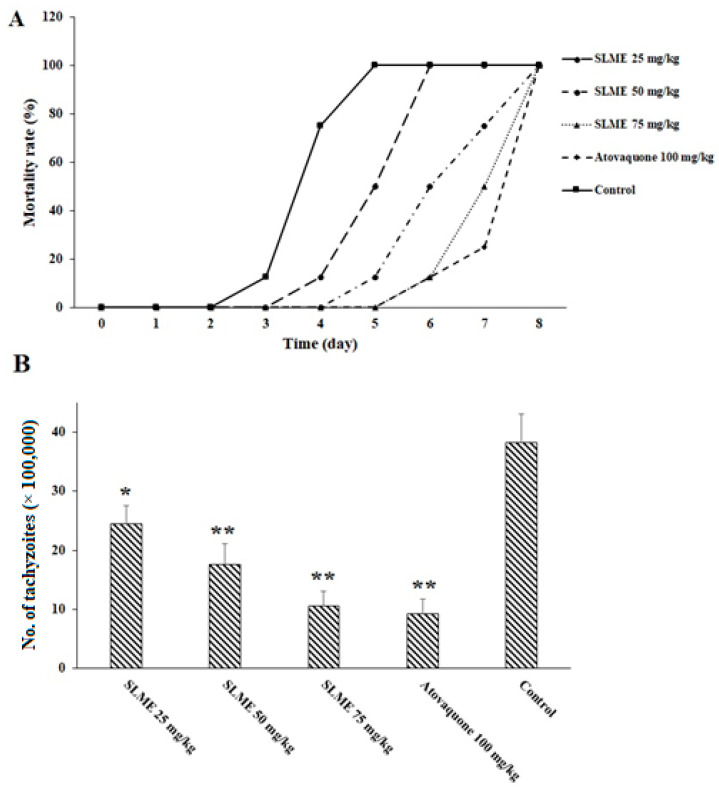
The mortality rate of mice (**A**) and the parasite load (at 1 mL of peritoneal exudate) on the 3rd day post-infection (**B**) receiving the SLME at the doses of 25, 50, and 75 mg/kg/day for two weeks compared with the control group (normal saline). * *p* < 0.01 and ** *p* < 0.001 compared to the control.

The in vivo efficacy of various doses of the SLME is revealed in Figure 4. The obtained findings exhibited that 2 weeks’ pre-treatment of mice with SLME at the doses of 25, 50, and 75 mg/kg/day elevated the survival rate of infected mice by the sixth, seventh, and eighth days, respectively. The parasite load of the obtained tachyzoites from mice in each tested group is also displayed in Figure 2. Based on the obtained results, after treatment of the mice with SLME at the doses of 25, 50, and 75 mg/kg/day, the number of tachyzoites on the third day considerably declined by 36.3, 54.6, and 72.3%, respectively. But, the number of parasites in the mice that received atovaquone decreased by 75.7%.

### 3.6. Effect on Oxidative Stress and Proinflammatory Cytokines

**Figure 5:**
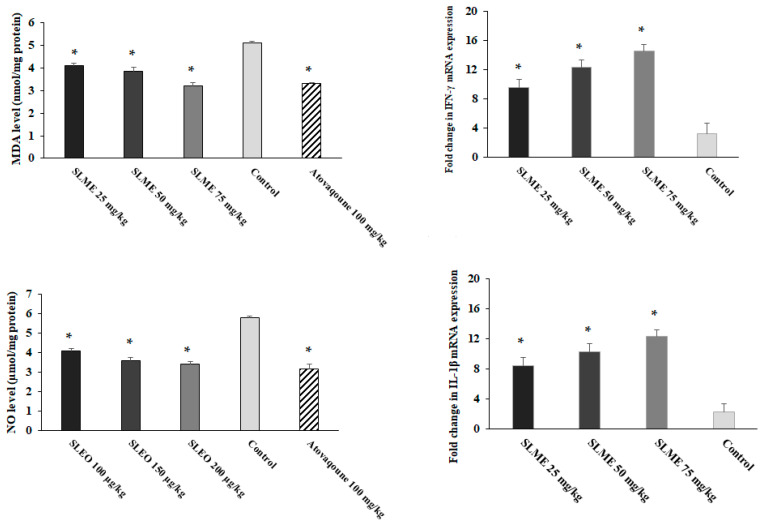
The level of MDA, NO, as well as IFN-γ and IL-1β expression levels in the infected mice (three mice from each group) receiving SLME at the doses of 25, 50, and 75 mg/kg/day for two weeks compared with the control group (normal saline). Results are displayed as the mean ± SD. * *p* < 0.01.

Based on the obtained results, although in control mice, the MDA and NO levels in liver tissue were considerably raised (Figure 5); but, the level of these factors was meaningfully (*p* < 0.05) decreased in mice pre-treated with SLME at the doses of 25, 50, and 75 mg/kg/day. Figure 5 showed that mice pre-treated with SLME at the doses of 25, 50, and 75 mg/kg/day triggered a considerable (*p* < 0.001) upregulation of IL-1β and IFN-γ mRNA gene expression levels in *T. gondii* mice on the third day after infection.

### 3.7. Toxicity Effects of SLME on Liver and Kidney Functions in Healthy Mice

**Figure 6:**
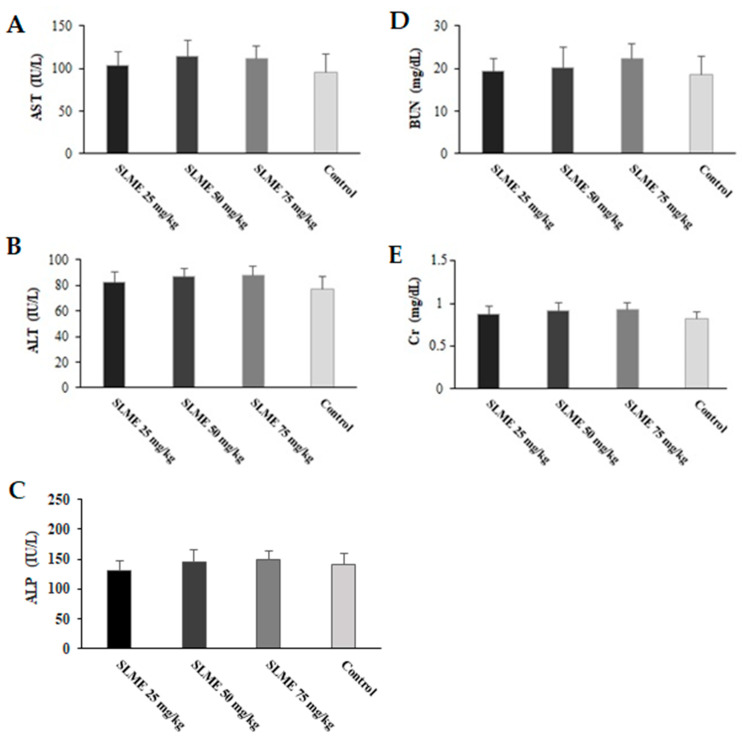
Effect of oral administration of SLME at the doses of 25, 50, and 75 mg/kg/day for 14 days on the serum level of AST, aspartate aminotransferase (**A**); ALT, alanine aminotransferase (**B**); ALP, alkaline phosphatase (**C**); BUN, blood urea nitrogen (**D**); and Cr, creatinine (**E**). Results are displayed as the mean ± SD.

As shown in Figure 6, no mortality was observed in mice that received the SLME at the doses of 25, 50, and 75 mg/kg/day for 2 weeks. According to the results, no significant change was observed in the serum level of BUN and Cr, as well as the activity of AST, ALT, and ALP in mice treated with SLME at the doses of 25, 50, and 75 mg/kg/day in comparison with the control group.

## 4. Discussion

Nowadays, the main treatment in people infected with toxoplasmosis is chemotherapy with a combination of pyrimethamine and sulfadiazine accompanied by other synthetic drugs such as atovaquone and spiramycin, etc. [7]. Since these synthetic drugs are associated with serious side effects, e.g., increasing the risk of bone marrow suppression, hematological toxicity, teratogenic effects, and renal complications [8], the discovery of effective drugs with minimal side effects and optimal effectiveness in the treatment of toxoplasmosis is necessary. The present study aimed to evaluate the efficacy and safety of the SLME against acute toxoplasmosis caused by the *T. gondii* RH strain in mice. The obtained results revealed that different concentrations of the SLME, especially at the concentrations of 150 and 75 µg/mL exhibited significant antiparasitic activity against *T. gondii* tachyzoites in vitro. By in vivo assay, we found that 2 weeks’ pre-treatment of infected mice with SLME at the doses of 25, 50, and 75 mg/kg/day declined the mean number of tachyzoites on the third day as well as reduced the mortality rate by the sixth, seventh, and eighth day after infection, respectively.

In recent years, the anti-*Toxoplasma* effects of various medicinal herbs with IC_50_ values ranging from 0.369 μg/mL to 1.87 mg/mL have been reported against the RH strain of *T. gondii* [8,28]. Considering the prophylactic effects of medicinal herbs on toxoplasmosis, in a study conducted by Kareshk et al. (2015), the results showed that oral administration of *Bunium persicum* essential oil at the concentrations of 0.05 and 0.1 mL/kg for 14 days, increased the life span of mice infected with the RH strain of *T. gondii* by days 6 and 7, respectively; whereas reduced the mean number of tachyzoites compared with the control group [29]. Mahmoudvand et al. (2020) showed that the oral administration of the *Zataria multiflora* essential oil at the doses of 0.2 and 0.4 mL/kg once a day for 14 days significantly increased the life span of mice infected with the RH strain of *T. gondii* by days 8 and 9, respectively, while a significant reduction in the mean number of tachyzoites in the mice was observed [30]. The results of another prophylactic study demonstrated that *Berberis vulgaris* extract at the dose of 1 and 2 g/kg, in addition to reducing the number of parasites, significantly increased the survival rate of NMRI mice infected by *T. gondii*, RH strain [31]. This discrepancy and variation in the results obtained in these studies compared to our study are probably due to some factors such as the extraction method, plant part used, solvent type, exposure times, type of animal, concentrations used, and variation in the amount of secondary metabolites present in these plants [32]. Recently, Barakat et al. [23] reported that propolis and wheat germ oil (WGO) showed a suitable combination of therapeutic efficacy against acute toxoplasmosis. The work revealed the potential synergistic effects of propolis and WGO on an herbal substance that could be considered an effective treatment of acute toxoplasmosis infection. These herbal substances reduced the parasite load significantly [33].

Considering the antiparasitic effects of *S. lavandulifolia*, Barati et al. (2017) have demonstrated that the aqueous and hexane extract of *S. lavandulifolia* had potent in vitro antiparasitic effects against *Giardia lamblia* cyst [34]. Sereshti et al. have revealed that watery and ethanolic extract of *S. lavandulifolia* at concentrations of 10–1000 μg/mL significantly reduced the viability of *Trichomonas vaginalis* trophozoites in vitro [35]. In a study conducted by Asadi et al., the results showed that hydroalcoholic extract of *S. lavandulifolia* at concentrations of 50 and 100 μg/mL displayed promising antileishmanial effects against promastigotes of *Leishmania major* in vitro [36].

In line with the previous investigations, we found the presence of some other secondary metabolites, such as glycosides, tannins, and terpenoids, was also confirmed in this extract [9,10]. Barakat et al. (2023) stated that propolis has many plant secondary metabolite contents (phenolic compounds) that work against many extracellular and intracellular protozoan parasites [33]. Reviews have demonstrated the antifungal, antiviral, antibacterial, and antiparasitic effects of flavonoids and phenolic compounds [37,38]. Regarding the antimicrobial mechanisms of flavonoids and phenolic compounds, previous studies confirmed that these compounds displayed their antimicrobial mechanisms through the suppression of nucleic acid synthesis, blockage of cytoplasmic membrane function, suppression of energy metabolism, prevent bacterial virulence factors, display of a synergistic effect with current synthetic drugs, etc. [38,39].

In addition to the antimicrobial mechanisms of these secondary metabolites, several studies revealed that flavonoids and phenolic compounds can fortify the immune system, mainly the cellular immune system, through the mammalian/mechanistic target of rapamycin (mTOR) pathway signaling activity, stimulating some immune cells (e.g., macrophages, and natural killer cells), regulation of cytokine excretion, phagocytosis, triggering of macrophages, and production of immunoglobulins [40,41]. Hence, it can be acclaimed that *S. lavandulifolia* displayed its prophylactic effects against toxoplasmosis through the direct effect on parasites and also indirect mechanisms, especially ones that reinforce the immune system, mainly the cellular immune system.

It has been reported that LPO, through the induction of cell membrane destruction and hepatotoxicity, is an important factor in the pathogenicity of *T. gondii* [42]. As previously shown, malonaldehyde (MDA), which is produced in the last step of lipid peroxidation, can be considered a valuable indicator for studying the LPO level in tissues [42]. Based on the obtained results, although in group Ex1 mice, the MDA and NO levels in liver tissue were considerably raised; but, the level of these factors was significantly (*p* < 0.05) decreased in mice pre-treated with SLME at the doses of 25, 50, and 75 mg/kg/day. These results, therefore, demonstrated that SLME could delay the inflammatory process and protect the liver.

Previous studies showed that the regulation of the production of proinflammatory cytokines IL-1β, IFN-γ, etc., during toxoplasmosis is one of the main factors that can cause the host to survive longer after induction of sepsis and infection [6,43,44]. Alnomasy (2021) showed that *Allium sativum* essential oil promotes the innate immune system, proinflammatory cytokines, inhibition of hepatic injury, etc., in mice with acute toxoplasmosis [20].

We found that pre-treatment of mice with various doses of the SLME triggered a significant (*p* < 0.001) upregulation of the mRNA gene expression of the IL-1β and IFN-γ mRNA, demonstrating that the pre-treatment of *T. gondii* infection mice with SLME can provoke the cellular immune system in the infected mice. Today, it has been proven that apoptosis is one of the most important ways that host cells can show to deal with pathogenic pathogens [45]. The obtained findings of flow cytometry revealed the early apoptosis after exposure with SLME at 32.5, 75, and 150 μg/mL was 0.640%, 0.48%, 1.23%, and 1.72%, respectively, whereas the late apoptosis after exposure with SLME at 32.5, 75, and 150 μg/mL was 27.5, 67.8% and 76.7%, respectively, indicating that SLME displayed anti-*Toxoplasma* effects through induction of apoptosis.

Today, the study of the function of some important and vital organs of the body, such as the liver and kidneys, by measuring the serum levels of enzymes related to their function is one of the best and most reliable ways to assess the toxicity of new drugs [46]. On the other hand, the most common technique for evaluating liver and kidney function is determining the activity of liver (e.g., ALT, AST, and ALP) and kidney (Cr and BUN) parameters. Our results exhibited that no significant change was observed in the activity of AST, ALT, ALP, as well as the level of BUN and Cr in mice treated with SLME at the doses of 25, 50, and 75 mg/kg/day in comparison with the control group; indicating that SLME displayed no toxicity on liver and kidney organs in mice.

## 5. Conclusions

The obtained results of the current study showed the relevant in vitro anti-*Toxoplasma* effects of SLME against *T. gondii*. Moreover, the results confirmed the promising in vivo effects of SLME, which improved the survival rate of infected mice and declined the parasite load in the infected mice. We also found that SLME provokes the innate immune system, modulates the proinflammatory cytokines, and inhibition of hepatic injury in infected mice. With all these descriptions, further surveys are required to support these findings and elucidate the possible mechanisms of action of this plant.

## Figures and Tables

**Table 1:** The phytochemical analysis of the *Stachys lavandulifolia* methanolic extract.

Phytochemical	Test	Presence
Flavonoids	Ammonia test, alkaline reagent test	+
Saponins	Frothing test	-
Terpenoids	Salkowski test	+
Tannins	1% gelatin and 10% NaCl solutions	+
Alkaloids	Mayer and Dragendorff’s reagents	+

+ Presence of the phtochemical; - lack of the phtochemical.

## Data Availability

All data generated or analyzed during this study are included in this published article.
